# Variability of the Sheep Lung Microbiota

**DOI:** 10.1128/AEM.00540-16

**Published:** 2016-05-16

**Authors:** Laura Glendinning, Steven Wright, Jolinda Pollock, Peter Tennant, David Collie, Gerry McLachlan

**Affiliations:** a The Roslin Institute and Royal (Dick) School of Veterinary Studies, University of Edinburgh, Edinburgh, Midlothian, United Kingdom; b Monogastric Science Research Centre, Scotland's Rural College (SRUC), Edinburgh, Midlothian, United Kingdom; University of Michigan

## Abstract

Sequencing technologies have recently facilitated the characterization of bacterial communities present in lungs during health and disease. However, there is currently a dearth of information concerning the variability of such data in health both between and within subjects. This study seeks to examine such variability using healthy adult sheep as our model system. Protected specimen brush samples were collected from three spatially disparate segmental bronchi of six adult sheep (age, 20 months) on three occasions (day 0, 1 month, and 3 months). To further explore the spatial variability of the microbiotas, more-extensive brushing samples (*n* = 16) and a throat swab were taken from a separate sheep. The V2 and V3 hypervariable regions of the bacterial 16S rRNA genes were amplified and sequenced via Illumina MiSeq. DNA sequences were analyzed using the mothur software package. Quantitative PCR was performed to quantify total bacterial DNA. Some sheep lungs contained dramatically different bacterial communities at different sampling sites, whereas in others, airway microbiotas appeared similar across the lung. In our spatial variability study, we observed clustering related to the depth within the lung from which samples were taken. Lung depth refers to increasing distance from the glottis, progressing in a caudal direction. We conclude that both host influence and local factors have impacts on the composition of the sheep lung microbiota.

**IMPORTANCE** Until recently, it was assumed that the lungs were a sterile environment which was colonized by microbes only during disease. However, recent studies using sequencing technologies have found that there is a small population of bacteria which exists in the lung during health, referred to as the “lung microbiota.” In this study, we characterize the variability of the lung microbiotas of healthy sheep. Sheep not only are economically important animals but also are often used as large animal models of human respiratory disease. We conclude that, while host influence does play a role in dictating the types of microbes which colonize the airways, it is clear that local factors also play an important role in this regard. Understanding the nature and influence of these factors will be key to understanding the variability in, and functional relevance of, the lung microbiota.

## INTRODUCTION

Within the past 5 years, a diverse array of bacteria has been detected in healthy lungs through the use of non-culture-based methods (1, 2). These bacterial communities are commonly referred to as the lung microbiota and are thought to originate predominantly from the upper respiratory tract (3, 4). The presence of particular bacterial communities in the lung has been associated with several human diseases, including cystic fibrosis (5), chronic obstructive pulmonary disease (6), bronchiectasis (7), and lung transplant rejection (8).

While variation in the microbial communities present in the human lung exists at both large and small scales, based upon the location of the bacteria within the lungs (9) and the host cell types present (10), intraindividual variation has been found to be significantly less than interindividual variation, indicating that each individual may play host to a specific lung microbiota (9).

The lung microbiota of healthy domestic sheep has previously been investigated using culture-based methods (11–14), but these studies have shown conflicting descriptions of the extent of lung colonization by bacteria. A study of pneumonic Bighorn sheep lungs found that, for most sheep studied, bacterial 16S rRNA gene amplification and sequencing was able to identify additional bacterial species which were not found by culturing (15). Previous studies have also examined the upper respiratory tracts of healthy sheep by culture-based methods (11, 12, 14, 16). These studies are highly varied in the types and proportions of microbes identified.

Previously, our group studied the composition of the lung microbiota in sheep pre- and postinfection with Pseudomonas aeruginosa (17). That study included the first description of the lung microbiota communities of healthy domestic sheep by next-generation sequencing. A diverse community of microbes was identified, and variability was seen to be high, both within and between animals. The variability of the healthy lung microbiotas at specific lung sites over time has not been reported for any animal, although serial sampling of nondiseased humans is planned as part of the Lung HIV Microbiome Project (LHMP) (18).

In the present study, protected specimen brush samples were collected from three spatially disparate segmental bronchi at three time points (baseline, 1 month, and 3 months) to examine the compositions and variability of the lung microbiotas in healthy domestic sheep. In addition, samples were also taken from a separate sheep from a greater number of respiratory tract locations to further explore the extent of spatial variability.

Such studies are fundamental to understanding the functional relevance of lung microbiota in health and disease in ruminants. Indeed, bacterial pneumonia is well recognized in cattle and sheep and is often associated with high morbidity and mortality. Notably, regional predilection is evident in that infection by Pasteurella occurs most frequently in the apical and cardiac lobes in both sheep (12, 19) and cattle (20, 21). Coinfections with other respiratory pathogens are commonplace; it is already well known that infection by Bordetella parapertussis and Mycoplasma ovipneumoniae can lead to more-severe disease caused by Mannheimia (Pasteurella) haemolytica (22–25), and there are well-recognized links to stressful events, such as housing or transport. As it is conceivable that changes in the lung microbiota may precipitate or associate with such events, it is vital to ground future disease-related studies on a firm basis of understanding normal variation in health. While the immediate focus of such studies relates to animal health, it is also important to acknowledge that sheep are frequently used as models for human respiratory research (26, 27) and that there is an ongoing need to highlight any comparative contrasts and consistencies as and when they arise.

## MATERIALS AND METHODS

### Animals and airway sampling.

Six 20-month-old Suffolk cross sheep were used in this study (Table 1) (5 females, 1 castrated male) and were housed indoors in pens for the trial duration. No animals had undergone bronchoscopic examination during the 4 months preceding the study. Animal procedures were subject to the Animals (Scientific Procedures) Act of 1986 and were approved by the Roslin Institute Animal Welfare and Ethics Committee.

**TABLE 1:** Sheep used in this study

Sheep ID	Gender	Mean wt (kg) ± SD	Mean rectal temp (°C) ± SD
2D618	Female	51 ± 3.1	39.0 ± 0.06
2S066	Male (castrated)	69 ± 2.6	39.6 ± 0.20
2D619	Female	59 ± 1.7	39.3 ± 0.20
2D620	Female	64 ± 4.6	39.1 ± 0.21
2D644	Female	65 ± 1.0	39.3 ± 0.06
2D645	Female	70 ± 2.0	39.4 ± 0.06

**FIG 1:**
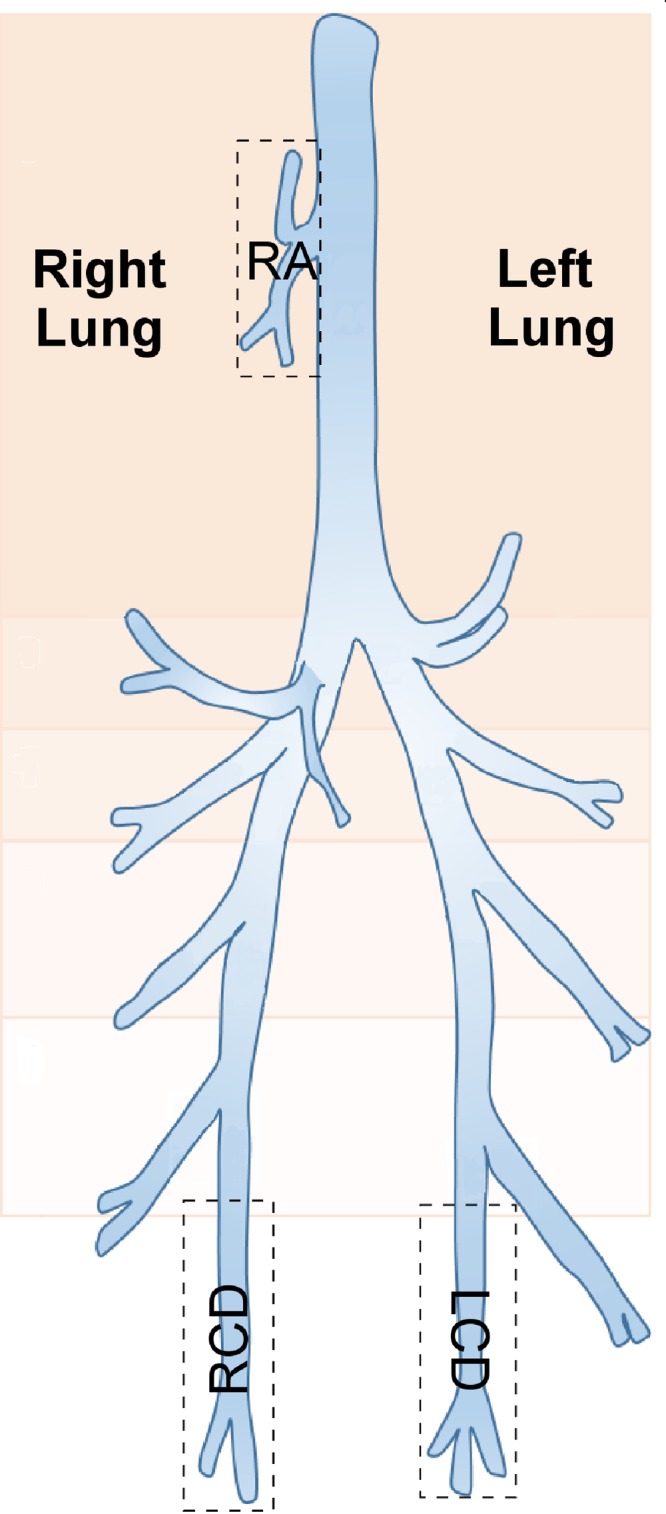
Diagram of a sheep lung, divided into anatomical segments. Boxes indicate the segments where protected specimen brushings were performed in the lungs of six sheep at three time points; these correspond to the right apical (RA), right caudal diaphragmatic (RCD), and left caudal diaphragmatic (LCD) segments.

Anesthesia was performed as described previously (28). Sheep were sampled by protected specimen brushings (disposable microbiology brush; ConMed, New York, NY, USA) at 0 days (baseline), 1 month, and 3 months. Sampling sites are shown in Fig. 1. Bronchoscopy was performed via an endotracheal tube by the same operator for all sheep at all time points. The sample harvest dates can be found in Table S1 in the supplemental material. Before sampling of every sheep on any given day, 7.5 ml of phosphate-buffered saline (PBS) was passed through the bronchoscope channel to act as an environmental quantitative PCR (qPCR) control. Bronchoscope washings were centrifuged at 13,000 × *g* for 15 min, and the pellet was resuspended in 500 μl of PBS.

**FIG 2:**
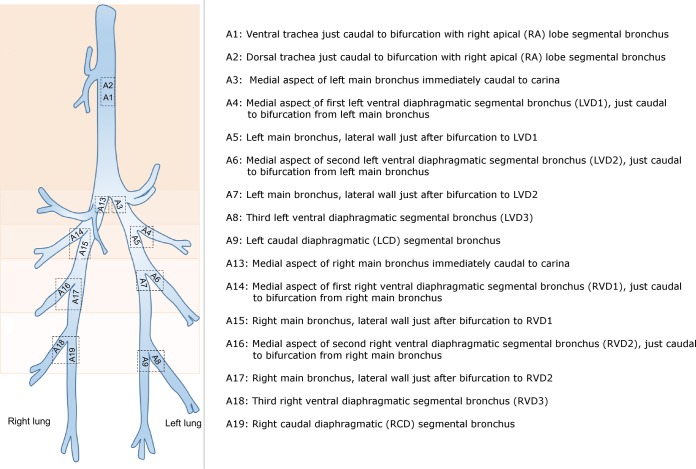
Locations of brushings within sheep lungs. Protected specimen brushings were performed in the sections of the lung labeled A1 to A9 and A13 to A19 in one sheep at one time point.

A throat swab and brushing samples (harvested as described above) were also taken from another sheep (female; age, 36 months; 60-kg body weight) at a single time point to further explore the spatial variability of the lung microbiotas (sampling date 1 May 2015). Brushing sites were dorsal and ventral trachea and paired sites from either side of airway bifurcations progressing along the anterior-to-caudal lung axis (Fig. 2).

### DNA extraction, amplification, and sequencing.

DNA extraction was performed using the Mo Bio (Carlsbad, CA, USA) PowerSoil DNA isolation kit. Brushes were transferred into PowerSoil bead tubes with PowerSoil solution C1 and PowerSoil bead solution. Bead tubes were heated at 65°C for 10 min and then placed in a FastPrep FP120 cell disrupter (Qbiogene, Inc., France) for 45 s at 5.0 m/s. From this point onward, the manufacturer's instructions were followed, except for the final elution step. Purified DNA was eluted into 50 μl rather than 100 μl of PowerSoil solution C6 to increase the DNA concentration.

All PCR steps used Q5 high-fidelity 2× master mix (New England BioLabs, Beverly, MA, USA). A nested PCR was performed with Illumina adaptor sequences and barcodes (see Table S2 in the supplemental material) included only on the primers for the second round in an attempt to reduce bias caused by barcoded primers when amplifying low-biomass samples (29). The conditions for the first round of PCR, amplifying the V1-to-V4 16S hypervariable regions (primers 28F [5′-GAGTTTGATCNTGGCTCAG-3′] and 805R [5′-GACTACCAGGGTATCTAATC-3′]), were as follows: 94°C for 2 min, followed by 20 cycles of 94°C for 1 min, 55°C for 45 s, and 72°C for 1.5 min, followed by 72°C for 20 min. The conditions for the second round of PCR, amplifying the V2-to-V3 16S hypervariable regions (primers 104F [5′-GGCGVACGGGTGAGTAA-3′] and 519R [5′-GTNTTACNGCGGCKGCTG-3′]), were as follows: 98°C for 30 s, followed by 20 cycles of 98°C for 10 s, 67°C for 30 s, and 72°C for 10 s, followed by 72°C for 2 min. Amplicons from both rounds of PCR were purified using the AMPure XP PCR purification system (Beckman Coulter, La Brea, CA, USA). Amplicons were sequenced using an Illumina MiSeq or HiSeq (Illumina, San Diego, CA) run producing paired-end 250-nucleotide reads (30). Those samples sequenced by two MiSeq runs are listed in Data Set S1 in the supplemental material, and those sequenced by HiSeq are listed in Data Set S2. When samples from the MiSeq runs were found to have low read numbers, they were sequenced again on a separate MiSeq run (samples 2D618 RA [right apical] at 3 months and 2D619 RA at 3 months). We previously confirmed cross-run stability by comparing separate runs made on the same samples (Fig. S1).

Extraction kit controls were produced by carrying out a reagent-only extraction using the Mo Bio PowerSoil DNA isolation kit. PCR reagent controls were constructed by adding 20 μl of nuclease-free water to the PCR mixture. The Human Microbiome Project Mock Community HM-782D (100,000 copies per organism per μl; BEI Resources, ATCC, Manassas, VA, USA), extraction kit controls, and PCR reagent-only controls and positive controls (DNA extracted from Pseudomonas aeruginosa strain PA0579) were amplified and sequenced by the same methods as were used for samples.

A separate mock community sample was sequenced using an Illumina HiSeq. For this sample, the solution produced from the first round of PCR was diluted 1:100 in nuclease-free water before being used in the second round of PCR. This was carried out to ascertain the effect on PCR bias of placing different concentrations of DNA into the second PCR round.

### Bioinformatic and statistical analysis.

Primers were removed using Cutadapt (31). Sequences which contained more than one base error per 10 primer bases were removed from further analysis. The following steps were carried out in mothur (32) and were based upon a protocol developed for MiSeq by the mothur creators (30). Forward and reverse reads were aligned to form one continuous DNA sequence; any sequences which failed to align were discarded. Sequences which contained ambiguous bases, were less than 369 bp in length, or contained homopolymers of greater than 9 bp were also discarded. Chimeras were identified and removed using UCHIME (33). Sequences were aligned to the SILVA reference alignment (34) and were classified using mothur's Bayesian classifier against the Greengenes database (35), which was trimmed to the V2-V3 hypervariable region of the 16S rRNA gene to improve classification depth (36). Sequences identified as not originating from bacteria were removed from further analysis. Operational taxonomic units (OTUs) were clustered into phylotypes using a database-dependent approach and then subsampled.

Distance matrices were created using Yue and Clayton theta values (37). Analysis of molecular variance (AMOVA) (38) was used to determine significant differences between the bacterial compositions of groups. Principal-coordinate analysis (PCoA) graphs were constructed to visualize similarities between samples. The inverse Simpson index was used to quantify diversity. Where data were nonparametric, the Friedman test was used to identify significant differences in diversity, using Minitab 16 for Windows (Minitab, Coventry, United Kingdom). All other statistical tests were carried out within mothur. Metastats (39) was used to identify OTUs which were different between groups. Good's coverage (40) was used to estimate sample coverage, and the Chao 1 index was used to calculate richness. Indicator OTUs (OTUs which are indicative of a particular group of samples) were identified using the indicator metric within mothur (41). Repeated-measures analyses of variance (ANOVAs) were carried out using the Vegan package in R (42–44).

### qPCR.

qPCRs were performed using the LightCycler 480 SYBR green I master mix (Roche Applied Science, Indianapolis, IN, USA), 1 μl of extracted DNA solution, and the 16S rRNA gene qPCR primers UniF340 (5′-ACTCCTACGGGAGGCAGCAGT-3′) and UniR514 (5′-ATTACCGCGGCTGCTGGC-3′) at a final concentration of 0.4 μM.

The qPCR run consisted of a preincubation step of 50°C (ramp rate, 4.80°C/s for 2 min) and then 95°C (ramp rate, 4.80°C/s for 10 s) and an amplification step consisting of 45 cycles of 95°C (ramp rate, 4.80°C/s for 30 s) and then 63°C (ramp rate, 2.50°C/s for 30 s). This was followed by a melting cycle consisting of 95°C (ramp rate, 4.80°C/s for 5 s) and then 65°C (ramp rate, 4.80°C/s for 1 min), followed by 97°C (ramp rate, 0.11°C/s; acquisition mode, continuous).

Negative controls consisted of both water and extraction kit reagent controls. For water controls, 1 μl of nuclease-free water was added to the qPCR mixture. For extraction kit controls, DNA extractions were carried out using the Mo Bio PowerSoil DNA isolation kit (Carlsbad, CA, USA) by following the same protocol as was used to extract DNA from samples, except that no sample was added, meaning that any bacterial DNA in the final elution must have been derived from the extraction kit reagents. Then, 1 μl of this elution was added to the qPCR mixture.

In order for us to compare the quantities of bacterial DNA found in bronchoscope wash and brushing samples, it was necessary to use a unit of measurement which could be applied to both sample types. Bacterial DNA concentrations are therefore reported as the 16S copy numbers present per microliter of eluent produced from samples by the Mo Bio PowerSoil DNA isolation kit. Statistical analysis was carried out in Minitab 16 for Windows. When data were nonparametric, the Mann-Whitney U test was used to statistically compare groups.

### Nucleotide sequence accession numbers.

The unassembled reads, with primers removed, are publicly available through the NCBI Sequence Read Archive (SRA) under the BioProject accession no. PRJNA298882.

## RESULTS

### Quality control and adequacy of sequencing.

After DNA sequences were constructed from the forward and reverse reads generated by sequencing, various quality control steps were performed to decrease the number of artifacts and poor-quality sequences used in subsequent analyses.

For the MiSeq runs, these steps resulted in a 15% loss of sequences (sequencing error rate = 0.39%). On average, samples contained 205,625 ± 27,232 (mean ± standard error of the mean [SEM]) sequences and a total of 925 bacterial OTUs were identified (see Data Set S1 in the supplemental material). Sequences were assigned to OTUs based on their taxonomic classifications. Each OTU does not necessarily represent an individual bacterial species but instead represents the lowest taxonomic level to which its bacterial sequences could be assigned. For example, 77.4% of reads could be identified to the genus level, while 31.1% could be assigned to the species level. If two species from the same genus could be assigned only to the genus level, then both were binned into the same OTU.

For the HiSeq run, samples contained on average 233,505 ± 69,735 (mean ± SEM) sequences, and the sequencing error rate was 0.39%. Six hundred thirty-three OTUs were identified (see Data Set S2 in the supplemental material), and the total reduction in sequence numbers due to quality control was 5%.

Good's coverage estimate values exceeded 97% for all samples. This indicates that at least 97% of the bacteria present in our original samples were likely to have been identified, demonstrating that the depth of sequencing was adequate.

Of the 20 bacteria contained in the mock community, all could be taxonomically identified down to genus level, except that Bacillus cereus, Escherichia coli, and Listeria monocytogenes could be identified only to the family level. This indicates that the primers were able to amplify a wide diversity of bacteria. While the proportions of bacterial DNA were different from the proportions anticipated if no PCR bias was present (Table 2), this was less apparent in the sample which had been diluted 1:100 after the first round of PCR. In the undiluted mock community, the proportions of bacterial orders differed from the expected proportions by an average of 9.48% (SEM, 2.24%; range, 0.99% to 19.48%), whereas the orders in the diluted mock community differed on average by 4.33% (SEM, 1.12%; range, 0.29% to 12.71%). This diluted mock community may be more comparable to the kind of biases we found in our samples, as the undiluted mock community contained a far higher concentration of template DNA (2,000,000 16S copies per μl) than our samples did on average (13,133 16S copies per μl).

**TABLE 2:** Proportions of DNA sequence reads belonging to bacterial members of a mock community

Taxonomy (order or genus)	Expected proportion of reads (%)	Actual proportion of reads (%)		Mock community species
		Undiluted	1:100 dilution	
Order				
Deinococcales	5	24.48	7.65	Deinococcus radiodurans
Campylobacterales	5	22.05	12.65	Helicobacter pylori
Bacteroidales	5	19.59	10.91	Bacteroides vulgatus
Bacillales	20	8.60	22.40	Bacillus cereus, Listeria monocytogenes, Staphylococcus aureus, Staphylococcus epidermidis
Lactobacillales	25	5.10	12.29	Enterococcus faecalis, Lactobacillus gasseri, Streptococcus agalactiae, Streptococcus mutans, Streptococcus pneumoniae
Clostridiales	5	4.01	7.86	Clostridium beijerinckii
Rhodobacterales	5	3.92	5.29	Rhodobacter sphaeroides
Pseudomonadales	10	3.42	5.97	Acinetobacter baumannii, Pseudomonas aeruginosa
Enterobacteriales	5	3.33	5.52	Escherichia coli
Neisseriales	5	2.17	3.49	Neisseria meningitidis
Actinomycetales	10	1.27	2.92	Actinomyces odontolyticus, Propionibacterium acnes
Other/unclassified	0	2.03	3.08	
Genus^*a*^				
Deinococcus	5	24.33	7.61	Deinococcus radiodurans
Helicobacter	5	22.04	12.65	Helicobacter pylori
Bacteroides	5	19.59	10.90	Bacteroides vulgatus
Rhodobacter	5	3.91	5.29	Rhodobacter sphaeroides
Clostridium	5	3.73	7.59	Clostridium beijerinckii
Staphylococcus	10	3.04	7.58	Staphylococcus aureus, Staphylococcus epidermidis
Lactobacillus	5	2.77	6.59	Lactobacillus gasseri
Pseudomonas	5	2.33	3.70	Pseudomonas aeruginosa
Neisseria	5	2.15	3.27	Neisseria meningitidis
Enterococcus	5	1.40	2.63	Enterococcus faecalis
Acinetobacter	5	0.97	1.62	Acinetobacter baumannii
Propionibacterium	5	0.76	1.77	Propionibacterium acnes
Actinomyces	5	0.48	1.12	Actinomyces odontolyticus
Streptococcus	15	0.47	1.63	Streptococcus agalactiae, Streptococcus mutans, Streptococcus pneumoniae
Other/unclassified	0	12.03	26.05	

a The species Bacillus cereus, Escherichia coli, and Listeria monocytogenes could not be classified to the genus level.

We assumed that PCR bias could reasonably be expected to apply equally across all samples and, therefore, that any statistical tests between samples should still be valid. The two bacterial species most overrepresented in the undiluted mock community (Deinococcus radiodurans and Helicobacter pylori) are not commonly associated with the respiratory tract, and bacteria from these genera were very rare within our data set.

### Longitudinal study in 6 sheep over 3 months.

**FIG 3:**
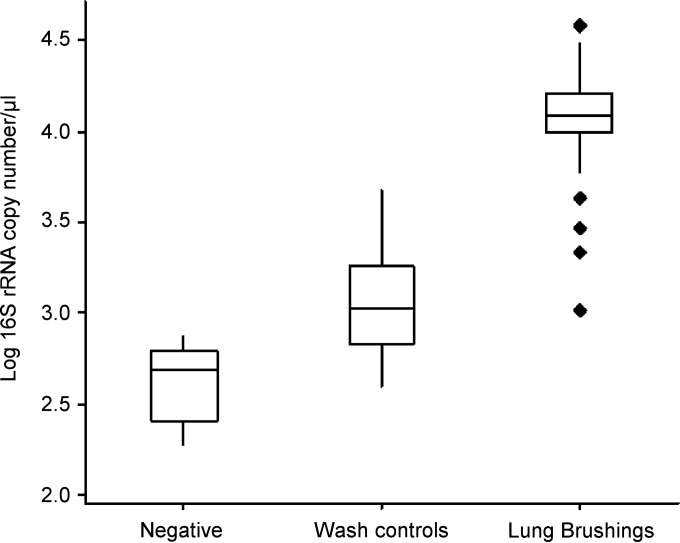
qPCR of lung brushing and control specimens. The bronchoscope channel was flushed with 7.5 ml of PBS, and the wash was collected (wash control, *n* = 18) prior to protected specimen brushing being performed on the lungs of sheep (lung brushings, *n* = 54). DNA was extracted from wash control and lung brushing specimens, and the quantity of bacterial DNA was calculated using 16S rRNA gene qPCR. Lung brushing specimens were found to contain significantly higher quantities of bacterial DNA than did wash controls (Mann-Whitney U test, *P* < 0.0001). Negative controls consisted of either water (*n* = 3) or extraction kit (*n* = 2) controls. Boxes, interquartile ranges; diamonds, outliers.

To examine the spatial, longitudinal, and interindividual variations of the sheep lung microbiota, lung brushing samples were taken from 3 spatially disparate lung locations (right apical [RA], right caudal diaphragmatic [RCD], and left caudal diaphragmatic [LCD]) in 6 sheep at 3 time points (baseline, 1 month, and 3 months). Estimates of total bacterial yield from qPCR analysis indicated that sheep lung brushing samples contained an average of 13,133 ± 894 (mean ± SEM) 16S copy numbers/μl (range, 1,032 to 37,627 16S copy numbers/μl). Bronchoscope wash control samples contained significantly lower bacterial 16S rRNA gene concentrations than lung brushing samples (Mann-Whitney U test, *P* < 0.0001), containing an average of 1,471 ± 279 (mean ± SEM) 16S copy numbers/μl (range, 397 to 4,792 16S copy numbers/μl) (Fig. 3). The qPCR-negative water controls were found to contain 190, 479, and 739 16S copy numbers/μl, and the extraction kit controls were found to contain 347 and 511 16S copy numbers/μl.

After sequencing and subsampling, bacterial communities isolated from the extraction kit and 16S PCR-negative controls were found to cluster separately from those found in sheep lung brushing samples (AMOVA, *P* < 0.001). Extraction kit controls were included from two different lots. The most abundant OTUs found in the first extraction kit control were Corynebacterium (36%), Enterobacteriaceae (13%), Mycobacterium llatzerense (7%), and Staphylococcus haemolyticus (5%). The most predominant OTUs in the second extraction kit control were Aerococcus (13%), Dermabacteraceae (11%), Micrococcus (10%), Enhydrobacter (9%), and Leuconostoc (7.2%). The predominant bacterial order present in both extraction kit controls was Actinomycetales (50.1% and 40.5%, respectively).

The bacteria isolated from lung brushing samples predominantly belonged to the orders Bacillales (26%), Actinomycetales (21%), Clostridiales (11%) and Lactobacillales (9%), while common genera included Staphylococcus (16%), Corynebacterium (9%), Jeotgalicoccus (5%), and Streptococcus (5%).

**FIG 4:**
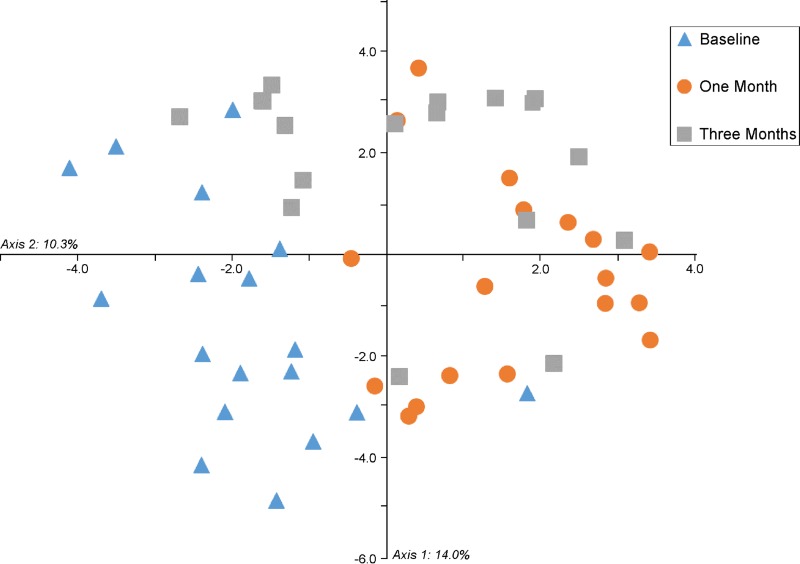
Clustering of time points by lung microbiota composition. A PCoA graph shows the similarities between bacterial communities sampled from three sheep lung segments in six sheep at three time points. Samples were found to cluster significantly by the time point at which they were taken (AMOVA, *P* < 0.001).

The underlying changes in bacterial OTUs between sampling points were examined. The bacterial communities found in lung brushing samples clustered significantly by time point (AMOVA, *P* < 0.001) (Fig. 4). The OTUs causing this clustering were identified by applying Metastats (see Tables S3 and S4 in the supplemental material). The largest difference observed between the first and second time points was an 11% increase in the abundance of an OTU identified as Corynebacterium. This is also the most abundant OTU in one of our extraction kit controls. OTU 12, Mycobacterium llatzerense, was also significantly more abundant at the 1-month time point and was the third-most-abundant OTU in the same extraction kit control. It therefore is likely that our time points were affected to different degrees by reagent contamination and that the analysis of segments over time is not possible. However, all samples taken in the same sheep at the same time point were processed using the same extraction kit; therefore, an analysis of spatial variability could be performed.

**FIG 5:**
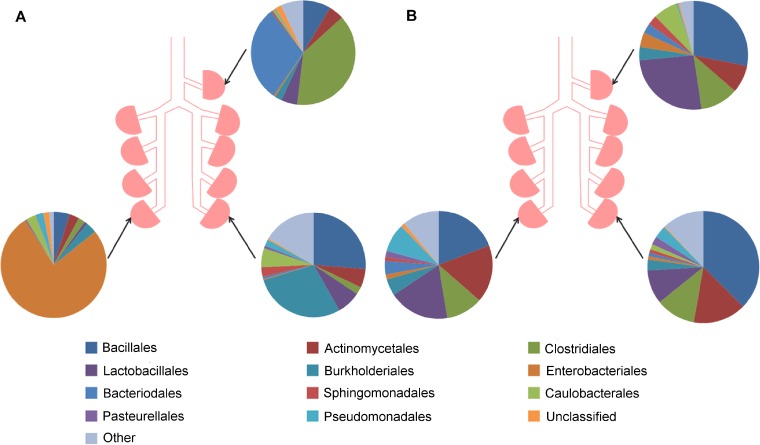
Bacterial communities found in three separate lung segments within two sheep. Protected specimen brushings were performed on the lungs of sheep at three different lung segments (RA, RCD, and LCD) at day 0. Sheep A (2S066) had highly different bacterial communities at each lung segment, whereas sheep B (2D644) had similar bacterial communities at all three lung sites.

Visual perceptions of community structure indicated that, in some sheep, samples taken from separate lung sites differed appreciably, whereas in other sheep, there appeared relative concordance between such samples (see the example shown in Fig. 5). A full visual summary of the results can be found in Fig. S2 in the supplemental material. There were no significant differences between the levels of diversity of communities located at different lung sites (inverse Simpson index, Friedman test, *P* > 0.5).

**FIG 6:**
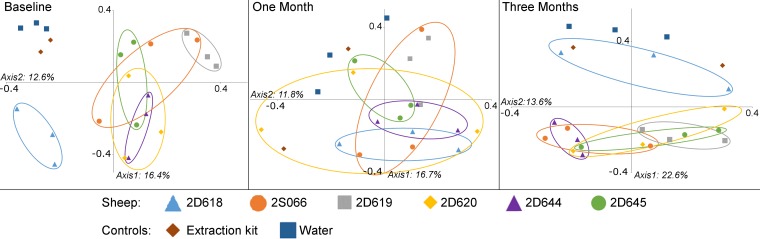
Clustering of individuals by lung microbiota composition. PCoA graphs show the similarities between the bacterial communities extracted from protected specimen brushing samples taken from sheep lungs at three time points (baseline [0 days], 1 month, and 3 months). Samples were taken from three separate lung segments (RA, RCD, and LCD). Samples from within the same sheep were found to cluster significantly at baseline (AMOVA, *P* = 0.001) and at 3 months (AMOVA, *P* = 0.045) but not at 1 month. This is likely to be due to the presence of contaminants originating from the extraction kits in the 1-month samples.

Sheep clustered separately by the compositions of their lung bacterial communities at the baseline time point (AMOVA, *P* = 0.001) and at the 3-month time point (AMOVA, *P* = 0.045), indicating that samples taken from within the same sheep were more similar to one another than to samples taken from other sheep. At the 1-month time point, sheep did not cluster in this manner (AMOVA, *P* = 0.394), though this is likely due to the presence of contamination causing a homogenization of our 1-month samples. Pairwise comparisons of samples showed no significant results. The similarity of samples to one another can be visualized using PCoA graphs (Fig. 6).

### Spatial variability of the lung microbiota in an individual sheep.

The observed variability between spatially disparate lung sites in some sheep prompted enquiry as to the consistency of bacterial communities sampled from sites in close spatial apposition.

Further samples were derived by systematically sampling multiple sites of the lungs of an individual animal at one time point. While the 3-month experiment did not include a control for every lot of extraction kit used, emerging literature and opinion within the field have since indicated the value of using the same extraction kit for all samples. This strategy, therefore, was adopted for these latter samples, which were all processed at the same time.

The extraction kit control was mainly composed of one OTU (OTU 18: 79%), which was also present in our brushing samples (mean ± SEM, 51.1% ± 3.3%). We felt confident in removing this OTU from all of our samples prior to analysis, as it could be identified to the species level (Methylobacterium komagatae) and was considered highly unlikely to be found within the sheep lung. No further OTUs were removed before analysis.

Lung brushing samples contained on average 2,116 16S copy numbers per μl (SEM, 365 copy numbers per microliter), while the throat swab and extraction kit control contained 42,480 and 43 16S copy numbers per microliter, respectively. The richness and diversity of the lung samples (Chao, 103.77 ± 7.32; inverse Simpson index, 14.24 ± 2.14) were found to be far lower than in the throat swab (Chao, 257.038; inverse Simpson index, 9.19). Sample A1, taken from the ventral aspect of the trachea just caudal to the bifurcation with the right apical lobe segmental bronchus, had the second-highest richness (Chao, 155.024) and diversity (inverse Simpson index, 8.713). However, sample A2, which was taken at the same level as sample A1 but from the dorsal aspect of the trachea, had much lower richness (Chao, 76.038) and diversity (inverse Simpson index, 4.925).

**FIG 7:**
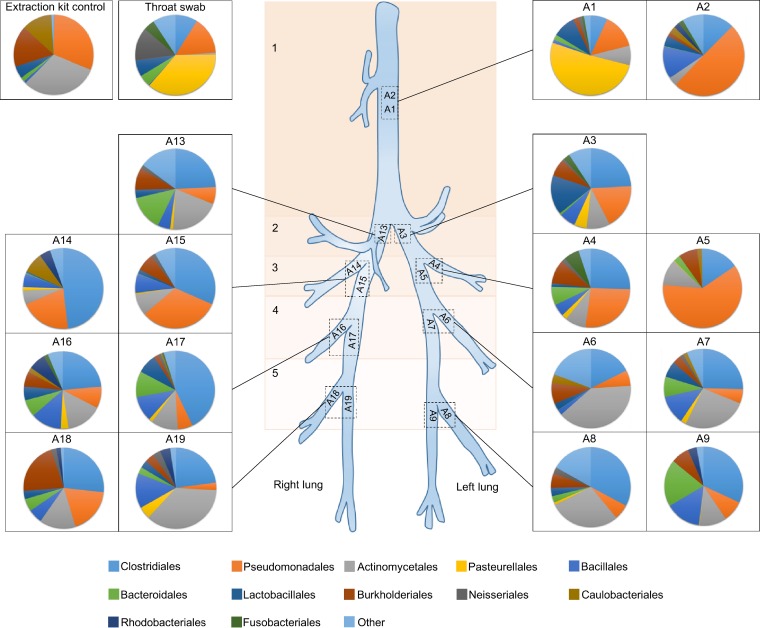
Diagram of the bacterial orders found in the sheep lung. Bacterial orders found in protected specimen brushing samples from the lung and trachea (A1 to A9 and A13 to A19), a throat swab, and an extraction kit control taken during a study of one sheep at one time point.

**FIG 8:**
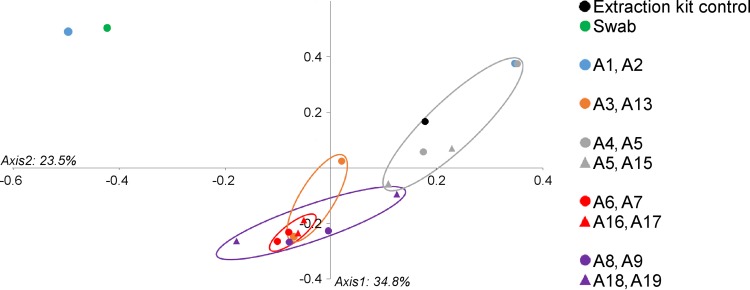
Clustering of lung brushing samples by depth within the lung. A PCoA graph shows the similarity of samples taken at different lung depths based upon the bacterial orders present. Lung depths are represented by color and correspond to different distances from the glottis, progressing in a caudal direction. Adjacent pairs of samples are represented by the same symbol and color. For the exact location of each sampling site, see Fig. 2. Subtracheal samples (≥A3) clustered significantly by lung depth (AMOVA, *P* = 0.033), as did paired samples (AMOVA, *P* = 0.046).

The compositions of the communities taken from the respiratory tract showed some variation, even between paired samples located very close to one another (Fig. 7). Subtracheal samples paired to their most proximate neighbor did not cluster together significantly when OTUs were defined at the lowest taxonomic depth (AMOVA, *P* = 0.30). However, paired samples did cluster significantly by the bacterial orders which they contained (AMOVA, *P* = 0.046). Subtracheal samples also clustered significantly (by order) based upon the depth in the lung from which samples were taken (AMOVA, *P* = 0.033) (Fig. 8) (lung depth in this context refers to increasing distance from the glottis, progressing in a caudal direction). An indicator OTU for the group which included the samples A4, A5, A14, and A15 was found to be OTU 4, Pseudomonadales (*P* = 0.042). The most abundant bacterial orders identified from brushings were Clostridiales (25.8%), Pseudomonadales (18.3%), and Actinomycetales (16.0%), while the throat swab was dominated by Pasteurellales (36.5%) and Pseudomonadales (15.1%). The extraction kit control was predominantly composed of Actinomycetales (31.1%) and Pseudomonadales (31.0%).

As the Pasteurellales order contains several species which are known to act as sheep lung pathogens and which display regional patterns of infection, we felt it would be interesting to investigate where OTUs belonging to this order were found within the respiratory tract (Table 3). By far, the largest proportion of these OTUs was found in the throat swab and in one of the tracheal brushing samples (sample A1).

**TABLE 3:** Abundances of the OTUs within the Pasteurellaceae family found in different locations of the sheep respiratory tract

Specimen type, location, or sample	% of organisms that were in:			
	OTU 5, Mannheimia	OTU 6, Pasteurellaceae	OTU 7, Bibersteinia	OTU 9, Bibersteinia trehalosi
Throat swab	23.7	10.1	1.8	0.7
Trachea				
A1	5.5	4.5	28.4	5.3
A2	0	0.03	0	0.01
Left lung				
A3	0	3.04	0.01	0.03
A4	1.2	0.006	0	0
A5	0	0.2	0.006	0
A6	0	0	0	0
A7	0.7	1.4	0.006	0.006
A8	0	0	0.8	0
A9	0.006	0.02	0.2	0
Right lung				
A13	0.9	0	0	0.006
A14	0.006	0.3	0	0
A15	0	0.6	0.006	0.006
A16	2.3	0.6	0.006	0
A17	0.10	0	1.3	0.01
A18	0	0.02	0.01	0
A19	3.2	0	1.3	0

## DISCUSSION

In order to better understand the variability present in the sheep lung microbiotas, we compared the lung bacterial communities of six sheep at three different lung sites over a duration of 3 months. To further explore the extent of spatial variability, we also took 17 samples from the respiratory tract of one sheep.

Previously, the bacteria in healthy domestic sheep lungs had been investigated by culture-based methods, which seemed to indicate that bacterial colonization of the sheep lung was rare or did not occur in all sheep (11, 12, 14). In contrast, using non-culture-based methods, we have found that all of the sampled sites in our seven sheep harbored diverse communities of bacteria, although in far smaller numbers than is generally found in other niches, such as the gut or upper respiratory tract.

Bacteria belonging to genera previously isolated from goat and sheep lungs (11, 12) were found in our samples. These included Corynebacterium, Bacillus, Enterococcus, Klebsiella, Mannheimia, Micrococcus, Moraxella, Pasteurella, Pseudomonas, Staphylococcus, and Streptococcus. Of the most common genera observed within our animals, Staphylococcus, Streptococcus, and Corynebacterium are commonly isolated from the upper respiratory tracts and skin of many animals, whereas Jeotgalicoccus is a less well-known genus (45) which has not been found to make up a substantial part of the lung microbiota communities in any previous studies. However, it has been isolated from the small intestinal mucosa of calves (46), the canine oral cavity (47), aerosol samples from a poultry house (48, 49), cattle teats (50), lamb meat (51), the rumen of cattle (52), and aerosol samples near a dairy (53).

The most common bacterial orders found in the sheep lung during the 3-month study were Bacillales, Actinomycetales, and Clostridiales. This agrees with the findings of a previous study carried out by our group, which examined the sheep lung microbiota before and after infection with Pseudomonas aeruginosa (17). Pseudomonadales (mainly Pseudomonas) was also commonly found in the lungs during our single-sheep study, while the throat swab from this study was dominated by Pasteurellales and Pseudomonadales.

Coinfection with Bordetella parapertussis or Mycoplasma ovipneumoniae has been shown to lead to more-severe disease caused by Mannheimia (Pasteurella) haemolytica (22–25). Mycoplasmas were very rare within our data set, with only one sheep segment containing reads from this genus at one time point. We did not identify any OTUs as Bordetella; however, we did find an OTU designated Alcaligenaceae (the family to which Bordetella belongs), though this was uncommon and occurred in low abundance. We identified several OTUs which were classified as members of the Pasteurellaceae family, including Mannheimia, Bibersteinia, and, less commonly, Aggregatibacter segnis, Haemophilus parainfluenzae, Bibersteinia trehalosi, and Actinobacillus parahaemolyticus. All of these microbes have previously been isolated from the lungs or upper respiratory tract (54–58). Despite the fact that disease caused by members of this family is often located in the apical and cardiac lobes (12, 19), we observed members of this family to be present across the lung.

The composition of the lung microbiota found in our sheep shows some differences from that previously identified in humans, where Bacteroidales are found in higher numbers and there are generally fewer members of the Actinomycetales and Clostridiales orders (2, 9, 59). Segal et al. identified various bacterial taxa that were commonly found in high relative abundance in human lungs (1). These included taxa which were found in all of our sheep samples in high relative abundance (Streptococcus, Staphylococcus, Corynebacterium), taxa which were found in the majority of our samples but in lower abundances (Propionibacterium, Pseudomonas), and taxa which were found only sporadically in our samples and were usually in low abundance (Stenotrophomonas, Prevotella, Veillonella, Fusobacterium, Porphyromonas).

Such differences may at least in part reflect the different surroundings in which sheep live, as well as behavioral or physiological features, such as rumination. A study using buccal swabs to identify bacteria originating from the rumen suggested that, as the time between regurgitation and sampling increases, the orally associated bacterial populations in the buccal cavity increase and the rumen-associated bacteria decrease, potentially contributing to interanimal variation (58). In future studies, it may be useful to take rumen and upper respiratory tract samples alongside lung samples to explore whether the variations between these sites and the lung are related.

Regardless of the highlighted differences between sheep and human lung microbiotas, there is a pressing need to understand the mechanisms that underlie the spatial and temporal variability of microbiota in the mammalian lung. These fundamental studies are difficult to facilitate in healthy human subjects as a consequence of the invasive nature of the repeated sampling protocol as well as the difficulty of controlling for the influence of environmental and/or lifestyle factors. Large-animal models can, however, play an important role in filling this need. Indeed, the physiological and immunological similarities between sheep and human lungs (60, 61) have contributed to the widespread use of sheep as translational models for human lung research (26, 27), including for asthma (62–65), the delivery of drugs via the upper respiratory tract (66–68), emphysema (69–71), pulmonary hypertension (72–74), physical lung injury (75–78), lung infection (28, 79–81), respiratory distress syndromes (82–85), asbestosis (86–88), and lung cancer (89, 90).

In our study, we examined the variability of the lung microbiota in sheep. Bacterial populations were often different between lung segments and between individuals, which confirms our previous observations (17). There was more similarity between samples from the same sheep at the baseline and 3-month time points than between samples taken from different sheep, but this was not found to be the case at the 1-month time point. Lung sample clustering by individuals has previously been identified in humans (9) and sheep (17).

Clearly, large differences can exist in the microbiota sampled from different lung segments at the same time point. This spatial variability of lung microbial populations can be observed in P. aeruginosa infections in cystic fibrosis patient lungs (91). The mechanisms underlying such observations have yet to be elucidated; however, possible candidate influences may include regional variability of physiological parameters, such as gas concentrations, osmolality, temperature, pH, and blood flow (92–96), which may lead to the creation of microhabitats providing a selective advantage to certain bacteria (97). It has previously been demonstrated that differences in pH can lead to changes in the colonic microbiota (98) and that temperature combined with humidity can lead to changes in the composition of the skin microbiota (99).

A longitudinal analysis of the lung microbiota at specific lung sites in healthy individuals has not previously been reported. Our goal was to define the variability of the lung microbiota over time and to detect whether there was a sheep lung microbiota “signature” which remains stable. Unfortunately, at the time of carrying out this study, the extent of the variability of bacterial DNA found within different lots of extraction kits was not yet known (100). While we, therefore, did include some extraction kit controls for our longitudinal study, we did not include controls for all lots which were used. Samples from different time points were also processed at different times. Due to our small sample sizes and the fact that samples clustered significantly by time point, we do not feel that accurate conclusions can be drawn about the temporal stability of the microbiota from our data. However, all samples taken from the same time point in the same sheep were processed at the same time. Therefore, we can be confident that the spatial variability that we observed within animals was not due to our methodology.

In some individuals, samples taken from different lung segments were found to be highly different from one another, whereas in others, the lung microbiota appeared to be quite stable across the lung sites. Another finding was the disappearance of the significantly separate clustering of sheep samples at the 1-month time point. This was correlated with an increase in the proportions of several OTUs found in sheep lungs, the most noticeable increase arising from an OTU classified as Corynebacterium, which was also the most abundant OTU in samples from one of our extraction kit controls. It is likely that the disappearance of significant clustering by individual at the 1-month time point is due to the increased presence of contamination in our samples.

OTUs that were identified in both samples and negative PCR and extraction kit controls were not removed from the analysis for the 3-month sheep study. The reason for this decision was that a number of bacteria commonly associated with the upper and lower respiratory tracts were present in these controls, including the genera Streptococcus and Pseudomonas, and it was judged that their removal would merely introduce another source of bias.

Equally, any specific *a priori* manipulation based around assumptions gleaned from the human literature regarding microbiota in the upper and lower respiratory tract are potentially ill advised. Indeed, it has been demonstrated that the microbes found in the lungs of animals often match those found in their bedding and hay (101). It is therefore not possible to dismiss environmental microorganisms as being due only to the contamination of samples.

In our spatial-variation study, one OTU was removed before analysis, as we felt confident that its presence was due to contamination of our extraction kit. Clustering of organisms in lung brushing samples by the lung depth from which they were taken was observed when OTUs were defined by bacterial order. Microorganisms in samples paired with their proximate neighbors were also found to cluster significantly separately from those in brushing samples taken elsewhere in the lung, but this may just be due to the fact that these samples were taken from the same lung depth. Certainly, further research to explore the relationship between lung depth and community composition appears warranted.

After sequencing a mock community of bacteria which contained equimolar concentrations of each bacterial species, we did find some bias present, with some bacterial species being overrepresented or underrepresented. These biases, which may be caused by various factors, including primer mismatching, PCR cycle number, and the bioinformatic pipeline used, are quite common in 16S sequencing (102–105). We also sequenced a 1:100 dilution of the same mock community and found that the apparent biases were far smaller. As the concentration of bacterial DNA in our samples was far lower than that of the undiluted mock community, we feel that the 1:100 dilution is likely to better represent the biases which may be present in our samples, as it is closer to their bacterial DNA concentrations. We believe that this vindicates our choice of DNA amplification strategy, including the use of nested PCR.

It may not be possible to claim that the bacterial abundances identified via 16S sequencing quantitatively represent the relative abundances of bacteria in the sample. Indeed, this is made even more difficult, as different bacterial taxa contain different copy numbers of the 16S rRNA gene (106). However, it seems logical to assume that, if the same methodology is used for all samples within a study, then the biases present will be the same for all samples, and therefore, comparisons between groups or claims about the types of microbes present in samples would still be valid.

In conclusion, we observed variability in sheep lung microbiotas both between and within individuals. In some animals, different lung segments contained highly different bacterial communities, whereas other animals showed similar communities at all lung sites. While spatial variation was observed to occur over both large and small distances across the lung, samples taken at the same lung depth clustered together separately from those taken at different lung depths. Further studies are needed to explore the stability of the healthy lung microbiota over time.

## Supplementary Material

Supplemental material
