# Supplementary material for: Variability of the Sheep Lung Microbiota
Source: Appl Environ Microbiol. 2016 May 16;82(11):3225–38. doi: 10.1128/AEM.00540-16 (PMC4959240; doi:10.1128/AEM.00540-16)
Supplement: Supplemental material [file supp_82_11_3225__index.html]

Supplemental material 

# Variability of the Sheep Lung Microbiota

## Supplemental material

- Supplemental file 1 -

  Comparison of bacterial communities found in lung brushings from a previous study (Fig. S1); bacterial orders found at three separate lung segments in six sheep at three time points (Fig. S2); dates of bronchial brushing samplings for six sheep at three time points. (Table S1); barcoded primer sequences used during the second round of PCR (Table S2); OTUs found to be significantly different between lung samples taken from sheep at the baseline and 1-month time points (Table S3); OTUs found to be significantly different between lung samples taken from sheep at the 1-month and 3-month time points (Table S4).

  PDF, 622K
- Supplemental file 2 -

  Bacterial OTUs, sequenced by Miseq, assigned to samples taken from sheep lungs and controls (Data Set S1).

  XLSX, 249K
- Supplemental file 3 -

  Bacterial OTUs assigned to samples taken from a sheep's respiratory tract and controls (Data Set S2).

  XLSX, 91K
