# Supplementary material for: Variability of the Sheep Lung Microbiota
Source: Appl Environ Microbiol. 2016 May 16;82(11):3225–38. doi: 10.1128/AEM.00540-16 (PMC4959240; doi:10.1128/AEM.00540-16)
Supplement: Supplemental material [file AEM.00540-16_zam999117158so1.pdf]

Supplemental material

Figure S1

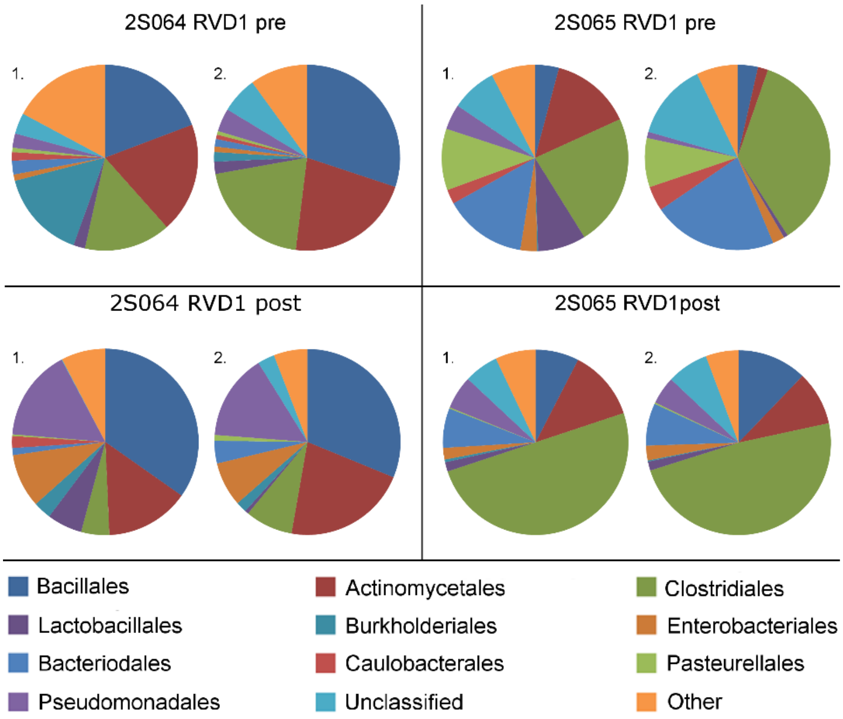

Comparison of bacterial communities found in lung brushings from a previous study (1). Protected specimen brushings were taken in two sheep (2S064 and 2S065) from the first ventral diaphragmatic segment of the right caudal diaphragmatic lobe (RVD1). Samples were taken pre and post injection with either colisitin (2S065) or saline (2S064). The bacterial DNA in these samples was amplified twice; once in May 2013 (1.) and once in September 2013 (2.). These amplicons were then sequenced and bioinformatically processed separately from one another. Despite some small differences, amplicons produced from the same sample are highly similar to one another.

Figure S2

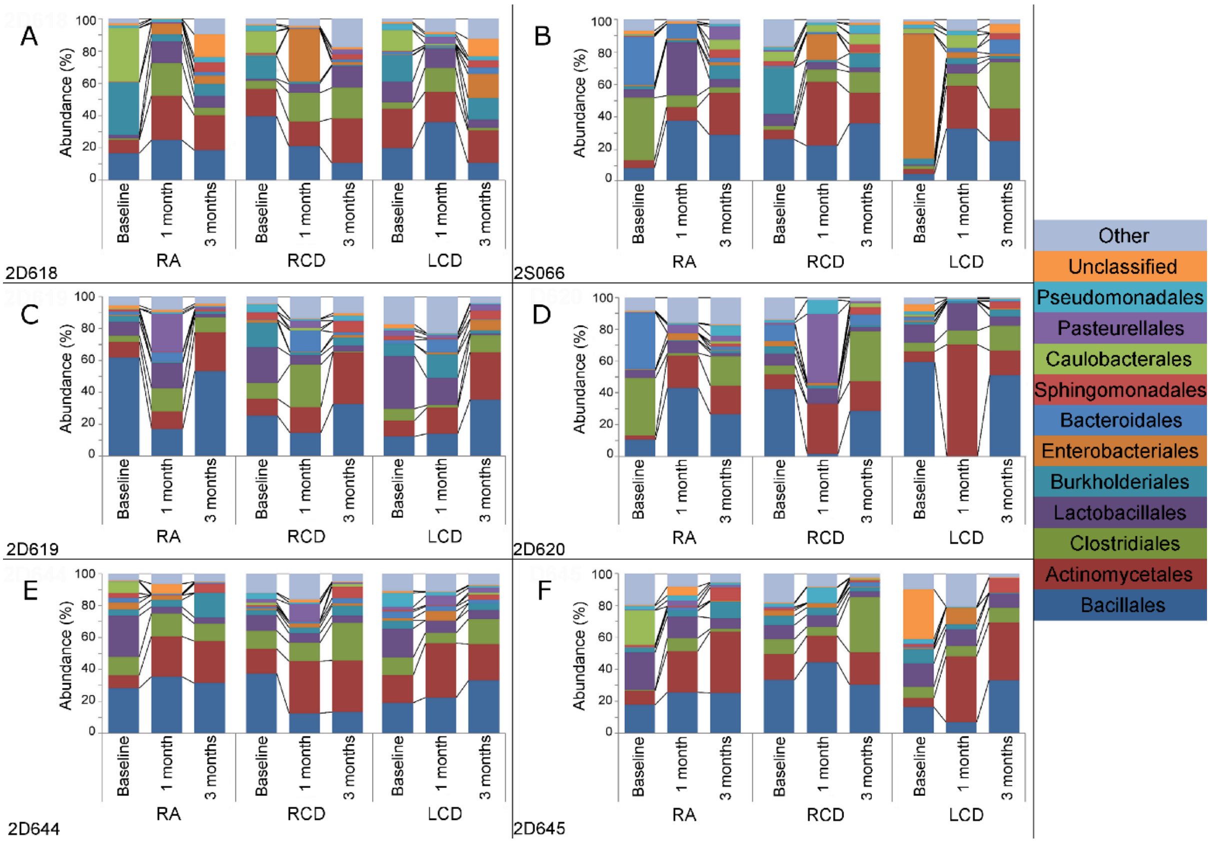

The bacterial orders found at three separate lung segments (RA: right apical, RCD: right caudal diaphragmatic and LCD: left caudal diaphragmatic) in six sheep (A: 2D618, B: 2S066, C: 2D619, D: 2D620, E: 2D644, F: 2D645) at three time-points: baseline (day 0), one month and three months.

Table S1: Dates of bronchial brushing samplings for six sheep at three time-points.

Time-point	Sheep	Date
Baseline (d0)	2D618	18/11/2013
	2S066	18/11/2013
	2D619	18/11/2013
	2D620	20/11/2013
	2D644	20/11/2013
	2D645	20/11/2013
One Month	2D618	16/12/2013
	2S066	16/12/2013
	2D619	16/12/2013
	2D620	17/12/2013
	2D644	17/12/2013
	2D645	17/12/2013
Three Months	2D618	17/02/2014
	2S066	17/02/2014
	2D619	17/02/2014
	2D620	18/02/2014
	2D644	18/02/2014
	2D645	18/02/2014

Table S2: Barcoded primer sequences used during the second round of PCR

Primer	Sequences
104f501	5'~AATGATACGGCGACCACCGAGATCTACACTATAGCCTACACTCT TTCCCTACACGACGCTCTTCCGATCTGGCGVACGGGTGAGTAA~3'
104f502	5'~AATGATACGGCGACCACCGAGATCTACACATAGAGGCACACTCT TTCCCTACACGACGCTCTTCCGATCTNGGCGVACGGGTGAGTAA~3'
104f503	5'~AATGATACGGCGACCACCGAGATCTACACCCTATCCTACACTCTT TCCCTACACGACGCTCTTCCGATCTNNGGCGVACGGGTGAGTAA ~3'
104f504	5'~AATGATACGGCGACCACCGAGATCTACACGGCTCTGAACACTCT TTCCCTACACGACGCTCTTCCGATCTNNNGGCGVACGGGTGAGTAA~ 3'
104f505	5'~AATGATACGGCGACCACCGAGATCTACACAGGCGAAGACACTCT TTCCCTACACGACGCTCTTCCGATCTGGCGVACGGGTGAGTAA~3'
104f506	5'~AATGATACGGCGACCACCGAGATCTACACTAATCTTAACACTCTT TCCCTACACGACGCTCTTCCGATCTGGCGVACGGGTGAGTAA~3'
104f507	5'~AATGATACGGCGACCACCGAGATCTACACCAGGACGTACACTCT TTCCCTACACGACGCTCTTCCGATCTGGCGVACGGGTGAGTAA~3'
104f508	5'~AATGATACGGCGACCACCGAGATCTACACGTACTGACACACTCT TTCCCTACACGACGCTCTTCCGATCTGGCGVACGGGTGAGTAA~3'
519r701	5'~CAAGCAGAAGACGGCATACGAGATATTACTCGGTGACTGGAGTT CAGACGTGTGCTCTTCCGATCTNNNGTNTTACNGCGGCKGCTG~3'
519r702	5'~CAAGCAGAAGACGGCATACGAGATTCCGGAGAGTGACTGGAGTT CAGACGTGTGCTCTTCCGATCTNNGTNTTACNGCGGCKGCTG~3'

519r703	5'~CAAGCAGAAGACGGCATACGAGATCGCTCATTGTGACTGGAGTT CAGACGTGTGCTCTTCCGATCTNGTNTTACNGCGGCKGCTG~3'
519r704	5'~CAAGCAGAAGACGGCATACGAGATGAGATTCCGTGACTGGAGTT CAGACGTGTGCTCTTCCGATCTGTNTTACNGCGGCKGCTG~3'
519r705	5'~CAAGCAGAAGACGGCATACGAGATTTCTGAATGTGACTGGAGTT CAGACGTGTGCTCTTCCGATCTGTNTTACNGCGGCKGCTG~3'
519r706	5'~CAAGCAGAAGACGGCATACGAGATACGAATTCGTGACTGGAGTT CAGACGTGTGCTCTTCCGATCTGTNTTACNGCGGCKGCTG~3'
519r707	5'~CAAGCAGAAGACGGCATACGAGATAGCTTCAGGTGACTGGAGTT CAGACGTGTGCTCTTCCGATCTGTNTTACNGCGGCKGCTG~3'
519r708	5'~CAAGCAGAAGACGGCATACGAGATGCGCATTAGTGACTGGAGTT CAGACGTGTGCTCTTCCGATCTGTNTTACNGCGGCKGCTG~3'

Table S3: OTUs found to be significantly different between lung samples taken from sheep at the baseline and one month time-points (Metastats: P = 0.001)

OTU phylotype	Relative abundance (%) mean ± SEM *	
	Baseline	One month
Bacteroides	0.69 ± 0.51	0 ± 0
Bradyrhizobium	0.19 ± 0.069	0.0051 ± 0.0051
Burkholderia	3.34 ± 1.69	0 ± 0
Burkholderia graminis	0.28 ± 0.15	0 ± 0
Comamonas	0.028 ± 0.0001	0 ± 0
Corynebacterium	0.72 ± 0.19	11.05 ± 3.54
Corynebacterium kroppenstedtii	0.0025 ± 0.0025	2.97 ± 0.57
Gallicola	0 ± 0	0.14 ± 0.095
Granulicatella	3.85 ±0.62	0.82 ± 0.42
MBA08	0 ± 0	0.12 ± 0.12
Methylobacteriaceae	0.22 ± 0.093	0 ± 0
Methylobacterium organophilum	0.12 ± 0.054	0 ± 0
Mycobacterium llatzerense	0.015 ± 0.009	2.47 ± 0.97
Roseburia	0.10 ± 0.081	0 ± 0
Ruminococcus	0.10 ± 0.081	0 ± 0

Sphingomonas yabuuchiae	0.78 ± 0.22	0 ± 0
Staphylococcus haemolyticus	0 ± 0	0.63 ± 0.63
Streptococcus infantis	1.51 ± 0.26	0.025 ± 0.02
Tannerella	0.12 ± 0.12	0 ± 0
Tepidimonas	0 ± 0	0.25 ± 0.25
Tetragenococcus	0.11 ± 0.11	0 ± 0

* OTUs are not included where they were less than 0.1% abundant at both time-points

Table S4: OTUs found to be significantly different between lung samples taken from sheep at the one month and three month time-points (Metastats: P = 0.001)

OTU phylotype	Relative abundance (%) mean ± SEM*	
	One month	Three months
Actinobacillus parahaemolyticus	0 ± 0	0.12 ± 0.11
Bacteroidales	0 ± 0	0.40 ± 0.39
Blastomonas	0.0051 ± 0.0051	1.5 ± 0.18
Blautia	0.20 ± 0.20	0 ± 0
Caulobacter	0 ± 0	0.21 ± 0.16
Cohnella	0 ± 0	0.11 ± 0.11
Comamonas	0 ± 0	0.76 ± 0.21

Helcococcus	0.23 ± 0.16	0 ± 0
MBA08	0.12 0.12	0 ± 0
Mycobacterium	0.048 ± 0.046	1.9 ± 0.46
Mycobacterium llatzerense	2.47 ± 0.98	0 ± 0
Myroides odoratimimus	0 ± 0	0.16 ± 0.16
Novosphingobium	0.018 ± 0.013	0.32 ± 0.096
Oligella	0 ± 0	0.11 ± 0.11
Prevotella copri	0 ± 0	0.18 ± 0.18
Prevotella tannerae	0 ± 0	0.12 ± 0.12
Rubricoccus	0.35 ± 0.25	0 ± 0
Schlegelella	0 ± 0	0.11 ± 0.11
Sneathia	0.20 ± 0.20	0 ± 0
Sphingobium yanoikuyae	0 ± 0	2.70 ± 0.31
Tepidimonas	0.25 ± 0.25	0 ± 0

* OTUs are not included where they were less than 0.1% abundant at both time-points

References

1. **Collie D, Glendinning L, Govan J, Wright S, Thornton E, Tennant P, Doherty C, McLachlan G.** 2015. Lung microbiota changes associated with chronic *Pseudomonas*

aeruginosa lung infection and the impact of intravenous colistimethate. PloS ONE **10:**e0142097. DOI: 10.1371/journal.pone.0142097
